# The Role of RamA on the Development of Ciprofloxacin Resistance in *Salmonella enterica* Serovar Typhimurium

**DOI:** 10.1371/journal.pone.0023471

**Published:** 2011-08-12

**Authors:** Yawei Sun, Menghong Dai, Haihong Hao, Yulian Wang, Lingli Huang, Yassir A. Almofti, Zhenli Liu, Zonghui Yuan

**Affiliations:** MOA Key Laboratory of Food Safety Evaluation/National Reference Laboratory of Veterinary Drug Residues (HZAU), Huazhong Agricultural University, Wuhan, Hubei, People's Republic of China; Indian Institute of Science, India

## Abstract

Active efflux pump is a primary fluoroquinolone resistant mechanism of clinical isolates of *Salmonella enterica* serovar Typhimurium. RamA is an essential element in producing multidrug resistant (MDR) *S.enterica* serovar Typhimurium. The aim of the present study was to elucidate the roles of RamA on the development of ciprofloxacin, the first choice for the treatment of salmonellosis, resistance in *S. enterica* serovar Typhimurium. Spontaneous mutants were selected via several passages of *S. enterica* serovar Typhimurium CVCC541 susceptible strain (ST) on M-H agar with increasing concentrations of ciprofloxacin (CIP). Accumulation of ciprofloxacin was tested by the modified fluorometric method. The expression levels of MDR efflux pumps were determined by real time RT-PCR. In ST and its spontaneous mutants, the *ramA* gene was inactivated by insertion of the *kan* gene and compensated on a recombinant plasmid pGEXΦ(*gst-ramA*). The mutant prevention concentration (MPC) and mutant frequencies of ciprofloxacin against ST and a spontaneous mutant in the presence, absence and overexpression of RamA were tested. Four spontaneous mutants (SI1-SI4) were obtained. The SI1 (CIP MICs, 0.1 mg/L) without any target site mutation in its quinolone resistant determining regions (QRDRs) and SI3 (CIP MICs, 16 mg/L) harboring the Ser83→Phe mutation in its QRDR of GyrA strains exhibited reduced susceptibility and resistance to multidrugs, respectively. In SI1, RamA was the main factor that controlled the susceptibility to ciprofloxacin by activating MdtK as well as increasing the expression level of *acrAB*. In SI3, RamA played predominant role in ciprofloxacin resistance via increasing the expression level of *acrAB*. Likewise, the deficiency of RamA decreased the MPCs and mutant frequencies of ST and SI2 to ciprofloxacin. In conclusion, the expression of RamA promoted the development of ciprofloxacin resistant mutants of *S. enterica* serovar Typhimurium. The inhibition of RamA could decrease the appearance of the ciprofloxacin resistant mutants.

## Introduction

*Salmonella enterica* serovar Typhimurium is considered as the main food-borne pathogen responsible for causing human disease [1]. Fluoroquinolones are the main drugs for the treatment of salmonellosis. However, the emergence of multidrug resistance (MDR) *S. enterica* serovar Typhimurium has led to the failure of the treatment [2], [3].

The resistant mechanisms of *S. enterica* serovar Typhimurium to fluoroquinolone mainly include target site mutations in quinolone resistant determining regions (QRDRs), decreased fluoroquinolone uptake and plasmid-mediated fluoroquinolone resistance [4]–[6]. Target site mutations in the QRDR of GyrA are very common and the mutations occur most frequently at codons Ser 83 and Asp 87. A single point mutation in the QRDR of GyrA can mediate high-level resistance to nalidixic acid and reduce susceptibility to fluoroquinolones. The GyrB mutations that have been identified are mostly located outside of the QRDR. The ParC mutations always occurred with mutations in GyrA and lead to high-level fluoroquinolone resistance [7]. MDR efflux pump is a membrane protein which can actively extrude drugs, dyes, disinfectants and detergents by the force of ATP hydrolysis or proton anti-direction movement. In the genome of *S. enterica* serovar Typhimurium, five of nine known MDR efflux pumps (AcrAB, MdtK, AcrEF, EmrB and MdfA) could extrude quinolones when overexpressed [8]. Most recently, active MDR efflux pump has been recognized as a primary fluoroquinolone resistant mechanism in clinical *S. enterica* serovar Typhimurium [9], [10].

Multiple efflux pumps in a single bacterial cell are often expressed under precise and elaborate transcriptional control. In *S. enterica* serovar Typhimurium, the regulators RamA, SoxS, MarA and AcrR have been reported to participate in the regulation of the expression of MDR efflux pumps [11], [12], [13]. When RamA was overexpressed in *S. enterica* serovar Typhimurium or *E.coli*, the strain exhibited decreased susceptibility to multidrugs [14], [15]. It has been confirmed that RamA can bind to the upstream promoter region of *acrAB* and *tolC* and increase the expression level of the efflux [16]. Recent study found that *ramR*, located in the upstream region of *ramA*, could repress the expression of *ramA* by binding to −10 region of its promoter [17]. In clinical isolates of MDR *S. enterica* serovar Typhimurium, amino acid mutations, deletions and frame shift mutations were also found in RamR [18].

Mutant prevention concentration (MPC) is the drug concentration that prevents the growth of first-step resistant mutants or the minimal inhibitory concentration of the most resistant organism present in the heterogeneous bacterial population when tested against greater than or equal to10^10 ^CFU/mL [19]. The MPC represents a threshold above which the selective proliferation of resistant mutants is expected to occur rarely. However, the strategy is based on the use of antibiotic concentrations that require bacteria to obtain two concurrent resistance mutations for growth [20]. Likewise, the range of antimicrobial plasma concentrations between the MIC and the MPC of wild bacterial population is defined as the mutant selection window (MSW) in which selective amplification of spontaneous drug-resistant mutants is more pronounced [21]. The concept of the MPC and MSW has been characterized in some main clinical pathogenic bacteria [22]–[25]. Recently, the MPCs and MSWs of different fluoroquinolones to clinical isolates of *S. enterica* serovar Typhimurium were reported [26]–[28].

Some reports demonstrated that active efflux pumps contributed in the development of ciprofloxacin resistance of *S. enterica* serovars Typhimurium [7], [29]. The regulator RamA played a predominant role in the development of MDR *S. enterica* serovars Typhimurium based on the fact that no MDR mutant was obtained when a susceptible strain with inactivated RamA was selected under the selection pressure of different drugs [30]–[31]. In addition to that, the RND efflux pump AcrAB which is an important efflux pump in MDR *S. enterica* serovars Typhimurium was shown to be mainly regulated by RamA [32], [33]. However, the overexpression of single AcrAB in the wild-type *S. enterica* serovar Typhimurium ATCC14028s lacking individual *acrAB* gene did not lead to the mutant exhibiting resistance to quinolones[8]. On the basis of the three-dimensional structure of AcrB, it captured the substrates from the periplasm or the outer leaflet of the cytoplasmic membrane [34], [35]. This might indicate the contribution of some single component efflux pumps in the fluoroquinolone resistance in *S. enterica* serovar Typhimurium. Furthermore, no study clearly showed whether RamA could activate the expression of some single component efflux pumps in *S. enterica* serovar Typhimurium. Likewise, the effect of RamA on the MPC value and MSW of *S. enterica* serovar Typhimurium to ciprofloxacin has not been reported until today.

The aim of this study was to elucidate the role of *ramA* in the development of ciprofloxacin resistance of *Salmonella enterica* serovar Typhimurium CVCC541 (ST). Spontaneous mutants were selected via several passages of *S. enterica* sreovar Typhimurium CVCC541 susceptible strain (ST) on M-H agar with increasing concentrations of ciprofloxacin. RamA was inactivated in ST and its spontaneous mutants and then, overexpressed on a recombinant plasmid. The MPCs and mutant frequencies of ST and a spontaneous mutant to ciprofloxacin in the presence, absence and overexpression of RamA were determined. Furthermore, the role of RamR, a repressor of RamA was also studied in a spontaneous mutant.

## Materials and Methods

### Drugs and reagents

Ciprofloxacin, ofloxacin, norfloxacin, sarafloxacin, enrofloxacin, chloramphenicol, and florfenicol were purchased from National Institute for the Control of Pharmaceutical and Biological Products (Beijing, China). Ampicillin, kanamycin, nalidixic acid, tetracycline, trypton and yeast extract were the products of Bio Basic Inc (BBI, America). Tetracycline and chloramphenicol, the better substrates for AcrAB [36] were used for determining MICs of *Salmonella enterica* serovar Typhimurium (CVCC 541) and the spontaneous mutants.

Unless otherwise indicated, the concentrations of ampicillin and kanamycin used in the present study were 80 and 50 mg/L, respectively.

### Bacterial strain and plasmids

*Salmonella enterica* serovar Typhimurium (CVCC 541), a clinical susceptible isolate from chicken in Changchun province in China was supplied by China Institute of Veterinary Drug Control (Beijing, China) and designed as ST in this report. The helper plasmids pKD46 and pKD4 were purchased from *E.coli* Genetic Stock Center in Yale University (New Haven, USA).

### Antimicrobial susceptibility testing

The susceptibility of ST and the selected mutants were tested using the two-fold broth microdilution method according to the CLSI guidance [37]. MIC values of all strains were determined on three independent occasions. *Escherichia coli* ATCC 25922 was used as the quality control in all the susceptibility tests.

### Selection of spontaneous mutants in vitro

Selection of spontaneous mutants with ciprofloxacin was performed as previously described with some modifications [38]. Briefly, ST was cultured in Luria-Bertani (LB) broth at 37°C overnight with vigorous shaking. 100 µL of the inoculum (10^9^ CFU/mL) was innoculated on MHA plates with increasing concentrations of ciprofloxacin (1×MIC, 2×MIC, 4×MIC, 8×MIC) and incubated for 24 to 48 h at 37°C. A single colony was selected from every selecting plate and incubated in LB broth containing the same concentration of the drug as that in the plate. The overnight culture of the colony from the selecting plate containing the highest concentration of ciprofloxacin was spread onto MHA plate supplemented with an increased concentration of ciprofloxacin. The selection procedure was repeated for the strains exhibiting high MIC of ciprofloxacin. The selected mutants in every procedure were passed 10 times in LB broth without antibiotics. MIC values of quinolones, tetracycline, chloramphenicol and florfenicol were examined and the selected mutants were stored in glycerol at −80°C until used.

### Amplification and Sequencing of the QRDRs

Genomic DNA from ST and the selected spontaneous mutants were extracted from overnight cultures in LB broth at 37°C by using the bacteria genomic DNA Mini extraction kit (Shanghai Generay Biotech Co. Ltd, China). The QRDRs of *gyrA*, *gyrB*, *parC* and *parE* were amplified using the primers (Table 1). The purified PCR products were sequenced at commercial company (Shanghai Sangon Biological Engineering Technology & Services Co. Ltd, China).

**Table 1:** Primers used in this study.

Primer	Primer sequence (5′-3′)	Product size (bp)	Reference
Primers used to inactivating the genes and verifying the mutants			
LRAF	ACA CGA TTG TCG AGT GGA TTG ATG ATA ATT TGA ATC AGC		17
	CGT TAC GTG TAG GCT GGA GCT GCT TC		
LRAR	ACG ATA AGC GCC TGG CGG CAGGTTGAACGTGCG GGT AAA		17
	AAT GCG CAT ATG AAT ATC CTC CTT AG	1567	
RAF	GAG CAC GAT GAC CAT TTC CG		
RAR	CCT GTC ATT CGC TTT ATC TGG	1679	this study
Primers used to testing target sites mutation			
GyrAF	CTA TCT GGA TTA TGC GAT GTC TGT C		
GyrAR	GAA CCG AAG TTA CCC TGA CCA T	273	this study
GyrBF	TGA ACG AAC TGC TGA GCG AAT A		
GyrBR	ATG GCT GGG CAA TGT AAA CG	540	this study
ParCF	TGG AAA ACG CCT ACT TAA ACT ACT C		
ParCR	GTA TTT GGA CAG GCG GGA TT	344	this study
ParEF	GTA CCG AGC TGT TCC TTG TG		
ParER	CCT TTC TTA CGC TTC AGT TGT T	436	this study
Primers used to real time RT-PCR			
16sF	GGT GTA GCG GTG AAA TGC GTA G		
16sR	CCA GGG CAC AAC CTC CAA GT	163	this study
AcrAF	AAA ACG GCA AAG CGA AGG TC		
AcrAR	ATG CGG GTT AGG GAA GAT GG	140	this study
MdtKF	CGT CGG CAT TTG TAT GGC TGT		
MdtKR	CAC GAC CTC AGG GTT GTC ATT G	94	this study
Primers used to amply the sequences of the regulatory loci			
RAPF	CCT TGA CGG CGT ATC TTT GC		
RAPR	GTC AAC GTG CGG GTA AAA ATG	495	this study
RARF	TGG CAG CCC TTG ATT ATG AG		
RARR	AGT GTT CGG TAA AAG GCA GTT C	797	this study
MARF	CTC CTA CCC ATC AGC GTT TCA		
MARR	ACA GGG CAG CAG CAT CAC AT	1236	this study
SOXSF	GCC GTT GGT TAC CGC TAT TA		
SOXSR	CGT TCA GTA TTG TCA GGG ATG G	660	this study
SOXRF	TAC ATA GCC CAG GTC CAT C		
SOXRR	TCG CTT ACA CTT ACA GTA TCA AC	983	this study
Primers used to the overexpression of RamA and RamR			
ERAF	GCG GGA TCC ATG ACC ATT TC		
ERAR	TTC TCG AGT CGC TTT ATC TGG C	402	this study
ERRF	TGG GAT CCC TTG ATT ATG AG		
ERRR	AGT CTC GAG TAA AAG GCA GTT C	797	this study

### Accumulation of ciprofloxacin

Accumulation of ciprofloxacin in ST and its spontaneous mutants in the presence or absence of CCCP (carbonyl cyanide-m-chlorophenylhydrazone, which dissipates the proton motive force and hence acts as an inhibitor of active efflux) or PAβN (Phenylalanine arginine beta-naphthylamide, which is a competitive inhibitor of resistance-nodulation-cell division [RND] pumps of gram-negative bacteria) was determined by a modified fluorometric method as previously described [9]. Fluorescence was measured with Infinite™ 200 microplate readers (TECAN Group Ltd., Austria) at excitation and emission wavelengths of 276 and 452 nm for ciprofloxacin, respectively. The amount of ciprofloxacin accumulated was calculated by comparison with a standard curve for ciprofloxacin (0.02 to 2 mg/L) in 0.1 M glycine hydrochloride (pH 3.0). Results were expressed as nanograms of ciprofloxacin incorporated per milligram (dry weight) of bacteria. All experiments were performed at least three times to ensure reproducibility. The mean and standard error was calculated.

### Examination of the expression levels of MDR efflux pumps

Total RNA from ST and its spontaneous mutants was harvested from 2 mL aliquots of culture using RNAprep pure Cell/Bacteria kit (TianGen BioTech Co. Ltd, China) according to the manufacturer's recommendation. DNA in total RNA was digested by RNase-free DNase (Promega, America). PCR amplification with the primers 16sF and 16sR (Table 1) was performed to confirm the complete DNA digestion. OD_260/280_ values of total RNA were detected using an Agilent 8451 UV-Visible spectrophotometer (Agilent Technologies, Palo Alto, USA).

Reverse transcription-PCR (RT-PCR) was performed using Superscript III reverse transcriptase (Invitrogen, America) according to the method described in the manufacturer. The synthesized cDNA was confirmed by PCR and stored at −20°C until used. Real-time PCR was performed using the IQ5 multicolour real-time PCR system (Bio-Rad, Hercules, USA) with specific primer pairs (Table 1), cDNA template and iQ SYBR Green Supermix (TakaRa Biotechnology Co. Ltd, Japan). PCR amplification was conducted with an initial step of 30 s at 95°C, followed by 35 cycles of 5 s at 95°C, 10 s at the annealing temperature (59°C for 16sRNA, *mdtK* for 61°C and *acrA* for 55°C) and 20 s at 72°C. To precisely test the relative expression level of the interesting genes, the standard curves of the amplification of 16sRNA, *mdtK* and *acrA* were individually established. CT values tested came within the linearity range for PCR amplification. Each sample was run at least twice independently. The 2^−Δ (Δ Ct)^ method was used to calculate increased fold of the gene tested in the mutants compared to that in ST.

### Inactivation and overexpression of the global regulator RamA

The *ramA* was inactivated in ST as previously described [39]. Briefly, kanamycin resistance gene *aph* flanked by FRT (FLP Recognition Target) sites was amplified by PCR under standard conditions using the template plasmid pKD4 and hybrid primers. These hybrid primers (Table 1) consisted of 20 nucleotides (nt) of the helper plasmid pKD4 and 45 nt on the 5′ and 3′ ends of the corresponding inactivated gene. The long PCR fragment (1567 bp) was purified, digested with *Dpn*I, repurified and transferred into ST by electroporation, in which the Red recombinase expression plasmid pKD46 was previously transformed. Transformants were selected on LB agar containing 25 mg/L of kanamycin at 37°C. The inserted sequence was amplified from intermediate kanamycin-resistant strain by using the primers which located outside of inactivated gene. The amplified PCR products were purified and sequenced. The mutant ST (*ramA::aph*) was designated as SR in this report.

The deletions were then transferred to the spontaneous mutants by P22HT105/int transductions as previously described [40]. The mutants SI1(*ramA::aph*), SI2(*ramA::aph*) and SI3(*ramA::aph*) were designated as SI1R, SI2R and SI3R, respectively.

The RamA was then compensated on a recombinant plasmid pGEXΦ(*gst-ramA*). Briefly, the recombinant plasmid pGEXΦ(*gst-ramA*) constructed with the vector pGEX-6p-1 and the sequence of *ramA* from ST was transformed into SR and SI2R by electroporation. The SR and SI2R strains harboring the recombinant plasmid pGEXΦ(*gst-ramA*) was designated as STRA and SI2RA, respectively. After the induction by IPTG, MICs of STRA and SI2RA to different antimicrobial agents and the expression level of *acrA* and *mdtK* in STRA were tested according to the above-mentioned methods.

### Amplification of the promoter regions of MDR efflux pumps and the sequence of regulators

The promoter regions of MDR efflux pumps (AcrAB and MdtK) and the sequences of the regulatory loci RamRA, MarRA, SoxRS and AcrR were amplified and sequenced. Sequence analysis was held using the following programs: BLAST (http://blast.ncbi.nlm.nih.gov/Blast.cgi) and the software DNAStar (DNASTAR Inc., Madison, WI).

### Compensation of RamR in SI1

The recombinant plasmid pET*his::ramR* was constructed from pET-28a vector and the sequence containing wild-type *ramR* from ST and transformed into SI1 by electroporation. The mutant harboring the recombinant plasmid pET*his::ramR* was designated as SI1RR. After the induction by IPTG, MICs of SI1RR to various antimicrobial agents were also determined as previously described.

### Growth curves of ST and SR

A single colony of ST or SR was cultured in 10 mL of Luria-Bertani (LB) liquid medium at 37°C without shaking for 24 h. OD_600_ values of the cultures were detected using an Agilent 8451 UV-Visible spectrophotometer (Agilent Technologies, Palo Alto, USA).The volume was adjusted slightly to give the same optical density at 600 nm for both strains. The adjusted culture was diluted 1:1000 into 300 mL of the same medium. 11 test tubes with 10 mL each from this culture medium were prepared and incubated at 37°C without shaking. Then 6 h later, a test tube was withdrawn and used to test the cells density of the cell culture at 600 nm. After that, a test tube was withdrawn every 3h, and the cell densities were monitored at OD_600_. Because the light scattering was not proportional to cell density above an OD_600_>1 at high cell densities, the OD_600_ was determined for a 1:10 dilution of the cultures. The growth curves of two strains were plotted in Microsoft Excel.

### MPCs and mutant frequencies of ciprofloxacin

For examination of the MPCs, the selected strains were individually grown overnight at 37°C with the vigorous shaking. Each overnight culture was diluted 1:100 into 20 mL of its corresponding liquid medium for another subculturing at the same condition. For the strain harboring the recombinant plasmid pGEXΦ(*gst-ramA*), when OD_600_ values of cell culture attained to 0.6, IPTG at a final concentration of 0.2 mM was added. After the culture for 8 h, all strains were centrifuged and concentrated by resuspending in 2 mL LB broth. 200 uL concentrated cell culture (approximately 10^10^ CFU) was plated on MH agar plates containing different concentrations of ciprofloxacin. Additionally, the inocula from each strain were plated onto antibiotic-free plates to obtain the precise number of CFU/mL. The antibiotic-containing plates were incubated at 37°C for 72 h, and the antibiotic-free plates were incubated under the same conditions for 24 h. Colony counts were conducted after 72 h incubation. The mutational frequencies of the strains tested to ciprofloxacin were calculated as the ratio of colonies grown on antibiotic-containing plates to colonies formed on antibiotic-free plates [27]. The MPC for each drug-isolate combination was defined as the lowest fluoroquinolone concentration that prevented growth of resistant mutants [21]. All MPC determinations were performed in duplicate. The MPC/MIC ratio was determined by dividing the MPC values by the MIC values.

## Results

### Resistant phenotype and target site mutations of spontaneous mutants

During stepwise selection with ciprofloxacin, four spontaneous mutants (SI1, SI2, SI3 and SI4) exhibiting decreased susceptibility to ciprofloxacin were obtained. Table 2 showed that SI1 without any target site mutations in its QRDRs exhibited decreased susceptibility to chloramphenicol, florfenicol, tetracycline as well as to quinolones. The target site mutation Ser83→Phe in the QRDR of GyrA in SI2 led to high-level resistance to nalidixic acid and decreased susceptibility to fluoroquinolones. SI3 harbored the same target site mutation as that in SI2. However, it exhibited fluoroquinolone resistance and showed significantly increase in the MICs of chloramphenicol, florfenicol, tetracycline compared to that of SI2. On the other hand, the MICs of SI4 to fluoroquinolones were higher than that of SI3. The second target mutation (Ala468→Glu) in QRDR of GyrB in SI4 might increase the resistance of the mutant to the tested fluoroquinolones.

**Table 2:** The susceptibility to antibiotics, the relative expression level of MDR efflux pumps and target sites mutations of the spontaneous mutants.

Strains	Antibiotic agents MICs (mg/L)									Target sites mutations		Fold change of the gene expression	
	CIP	NOR	OFX	SAR	ENR	NAL	TET	CHL	FLO	GyrA 83	GyrB 468	*acrA*	*mdtK*
ST	0.0125	0.03	0.05	0.03	0.04	6.25	6.25	3.13	3.13	No	No	1	1
SI1	0.1	0.4	0.4	0.2	0.4	10.42	12.5	10.42	12.5	No	No	6.08±2.44	3.87±0.78
SI2	0.4	0.8	1.6	1.4	0.8	>500	7.81	5.47	5.47	Ser→Phe	No		
SI3	16	53.33	32	26.67	21.33	ND	50	50	50	Ser→Phe	No	30.1±6.87	8.15±1.77
SI4	32	64	53.33	64	42.67	ND	33.33	50	50	Ser→Phe	Ala→Glu		
SR	0.0125	0.05	0.08	0.05	0.05	6.25	5.21	2.08	4.17				
SI1R	0.0125	0.1	0.2	0.05	0.05	6.25	3.1	3.1	6.25				
SI2R	0.3	0.8	1.6	1.3	0.8	>500	6.25	3.13	3.91	Ser→Phe			
SI3R	2	16	8	10.67	5.33	>500	6.25	6.25	8.33	Ser→Phe			
STRA	0.05	0.2	0.4	0.13	0.2	25	12.5	12.5	16.67			30.1±7.12	3.92±0.39
SI2RA	0.8	3.2	1.6	1.07	0.2	31	25	50	3.13	Ser→Phe			
SI1RR	0.025	0.2	0.2	0.1	0.1	12.5	6.25	6.25	12.5				

Notes: CIP, ciprofloxacin; NOR, norfloxacin; OFX, ofloxacin; SAR, sarafloxacin; ENR, enrofloxacin; NAL, nalidixic acid; TET, tetracycline; CHL, chloramphenicol; FLO, florfenicol; ND, not tested; No, no mutatation; ST, *Salmonella* Typhimurium (CVCC541); SI1-SI4, the spontaneous mutants derived from ST under the selection pressure of ciprofloxacin; SR, SI1R, SI2R and SI3R, the mutant of ST, SI1, SI2 and SI3 with inactivated *ramA*, respectively; STRA and SI2RA, SR and SI1R with the overproduction of RamA, respectively; SI1RR, SI1 with the overproduction of RamR.

### Effect of efflux pump inhibitors on the concentrations of ciprofloxacin accumulated in SI1 and SI3 compared to that in ST

**Figure 1:**
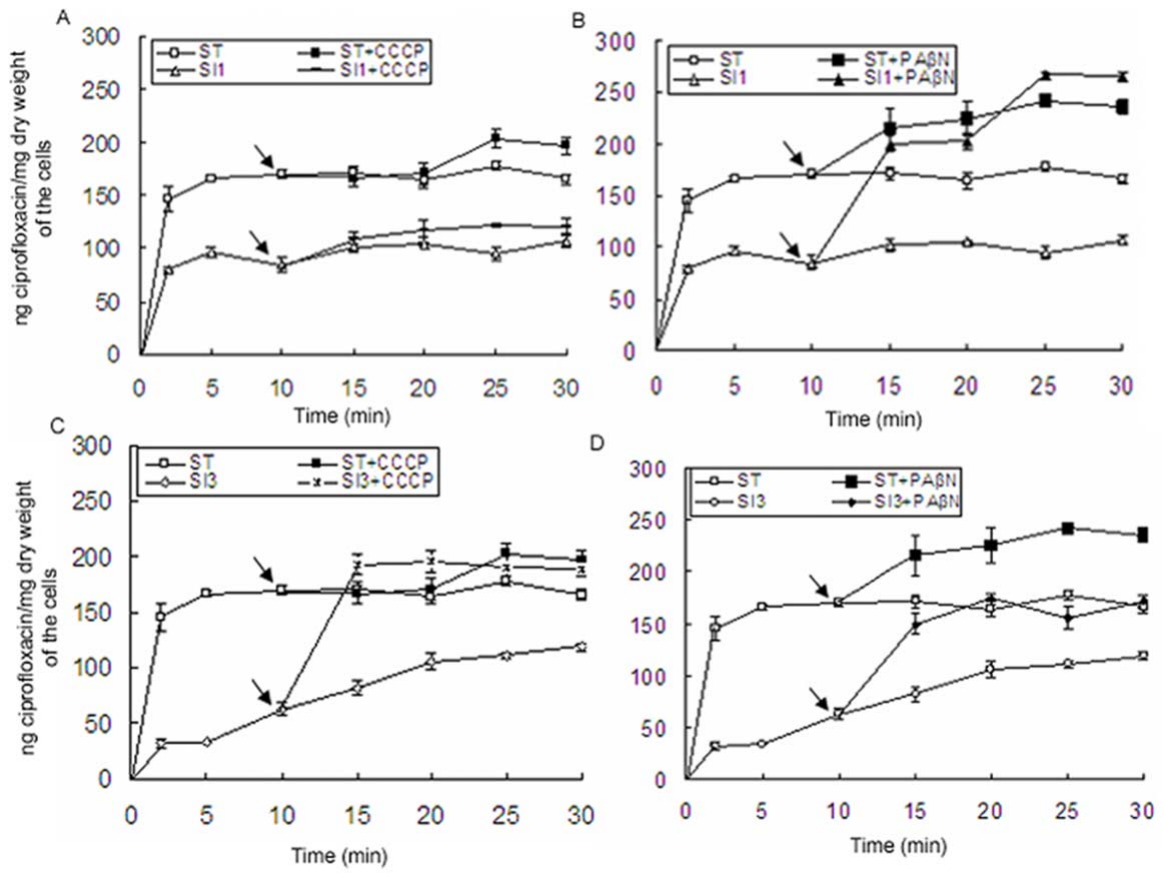
Accumulations of ciprofloxacin in the strains. Ciprofloxacin (10 mg/L) was added to each bacterial suspension at time zero; CCCP (100 µM) or PAβN (80 mg/l) was added at 10 min as indicated by the arrow; The SI (CIP MICs, 0.1 mg/L) and SI3 (CIP MICs, 16 mg/L) strains were the spontaneous mutants from ST under the selection pressure of ciprofloxacin; Each experiment represents the mean ± standard error of the mean of three separate experiments.

The spontaneous mutants SI1 without any target site mutation in its QRDRs and SI3 harboring the Ser83→Phe mutation in the QRDR of GyrA exhibited decreased susceptibility and high resistance to the tested drugs, respectively. Therefore, the concentrations of ciprofloxacin accumulated in the two strains compared to that in ST in the presence and absence of CCCP or PAβN were tested. The amounts of ciprofloxacin accumulated in SI1 and SI3 appeared to be lower than that in ST in the absence of efflux pump inhibiters (Figure 1). After CCCP was added, the amounts of the drug accumulated in SI1 slightly increased (approximately 1.15-fold), whereas lower than that in ST in the absence of CCCP [Figure 1(A)]. When CCCP was added into the incubation system containing SI3, the amount of ciprofloxacin accumulated in SI3 dramatically increased and was near to that in ST in the presence of CCCP [Figure 1(C)]. After the addition of PAβN, the accumulation of ciprofloxacin in SI1 increased and was near to that in ST in the presence of PAβN [Figure 1(B)]. Nevertheless, the concentration of ciprofloxacin accumulated in SI3 in the presence of PAβN was near to that in ST, whereas lower than that in ST in the presence of PAβN [Figure 1(D)].

### Role of RamA in the susceptibility of SI1to the tested drugs

In order to elucidate the role of RamA, the *ramA* in ST and SI1 was inactivated. Table 2 showed that the susceptibility of the SR (ST*ramA*::*aph*) strain to the tested drugs did not dramatically change compared to that of the ST. However, the MICs of SI1R (SI1*ramA*::*aph*) to the tested drugs except for nalidixic acid decreased 2- to 8-fold compared to that of SI1. The MICs of SI1R to ciprofloxacin, sarafloxacin, enrofloxacin and nalidixic acid were the same as that of SR. On the other hand, when RamA was overexpressed in SR, MICs of STRA to the tested drugs increased 2- to 6-fold compared to that of SR (Table 2). The MICs of STRA to ofloxacin and tetracycline were the same as that of SI1. The MICs of STRA to nalidixic acid was higher than that of SI1. The MICs of STRA (SR with the overproduction of RamA) to the remaining antimicrobial agents did not significantly change except that the MICs of STRA to ciprofloxacin, norfloxacin and enrofloxacin exhibited 2-fold decrease compared to that of SI1. Likewise, the results of real-time RT-PCR showed that the expression level of *acrA* and *mdtK* in SI1 and SI3 increased 6.08-, 3.87-fold and 30.1-, 8.15-fold, respectively, compared to that in ST. The expression level of *acrA* in STRA was similar to that in SI3 and MdtK expression in STRA was similar to SI1 (Table 2). As a result, RamA was the main factor that controlled the susceptibility of SI1 to ciprofloxacin by activating MdtK as well as increasing the expression level of *AcrAB*.

### Role of RamA in the susceptibility of SI3 to the tested drugs

When RamA was inactivated in SI3, MICs of SI3R (SI3*ramA*::*aph*) to the tested drugs except for nalidixic acid exhibited 2- to 8-fold decrease compared to that of SI3. The MICs of SI3R to ciprofloxacin decreased 8-fold (CIP: SI3, 16 mg/L; SI3R, 2 mg/L), which indicated that RamA played a predominant role in the ciprofloxacin resistance of SI3. However, the SI3R strain still exhibited resistance to the tested fluoroquinolones. In order to further elucidate the contribution of single RamA to the ciprofloxacin resistance of SI3, RamA was overexpressed in SI2R (SI2*ramA*::*aph*). Table 2 showed that the SI2R and SI2 strains had similar susceptibility to the tested drugs. When RamA was overexpressed in SI2R, the MICs of SI2RA to ciprofloxacin, norfloxacin, tetracycline and chloramphenicol increased 2-to16-fold compared to that of SI2R. The susceptibility of SI2RA to ofloxacin, sarafloxacin, and florfenicol was similar to that of SI2R. Unexpectedly, the susceptibility of SI2RA to enrofloxacin and nalidixic acid both exhibited reduced MICs compared to that of SI2R. To be noticed, the MICs of SI2RA to ciprofloxacin was lower than that of SI3R. The above-mentioned results demonstrated that some other efflux pumps not regulated by RamA contributed in the ciprofloxacin resistance of SI3.

Tetracycline and chloramphenicol were the good substrates of AcrAB-TolC [36]. When RamA was inactivated in SI3, the susceptibility of SI3R to tetracycline was the same as that of ST. When RamA was overexpressed in SI2R, the MICs of SI2RA to chloramphenicol was the same as that of SI3. Likewise, the expression level of *acrA* in SI3 was similar to that in STRA with the overproduction of RamA. These results indicated that the expression of RamA was mainly responsible for increasing the expression level of *acrAB* in SI3.

### Role of RamR in the susceptibility of SI1 to the tested drugs

The promoter regions of *acrAB* and *mdtK* in SI1 and SI3 were the same as that in ST (primers not shown in Table 1). No mutation was found in the sequence of regulatory loci MarRA, SoxRS and AcrR from SI1 and SI3. Likewise, the promoter region and open reading frame (ORF) of *ramA* in ST, SI1 and SI3 was the same. However, a nucleotide mutation (G499A) and a nucleotide deletion (C500) in the ORF of *ramR* from SI1 and SI3 were observed (the number of the nucleotide position starts from the initiation codon GTG).The nucleotide deletion led to the pro-formation of stop codon (TGA) at the position 562.

Whether the changes in RamR contributed in ciprofloxacin resistance by increasing the expression level of *ramA*, the complement of RamR was conducted in SI1. Table 2 showed the decreased susceptibility of SI1RR to the tested drugs except for nalidixic acid and florfenicol compared to that of SI1. The MICs of SI1RR to tetracycline reverted to that of ST. Although the MICs of SI1RR to the tested fluoroquinolones decreased 2-to 4-fold compared to that of SI1, the susceptibility of SI1RR did not revert to that of ST.

### Effect of RamA on the growth characteristic of ST

**Figure 2:**
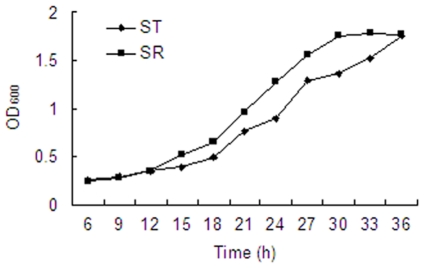
Growth curves of ST and SR (ST*ramA*::*aph*).

The growth speed of ST and SR did not vary during the culture period of the first 12 h (Figure 2). When the two strains entered into the logarithmic phase, the growth speed of SR was significantly higher than that of ST. The SR strain entered into the steady state phase after culturing for 30 h. However the ST strain attained the steady state phase after culturing for 36 h. The number of the bacteria in the initial and the steady state phase of both strains were confirmed by plating serial dilutions in MH agar without drugs. The initial concentration of both strains was 10^5^ CFU/mL. While when they reached the steady state phase, the concentration of the strains was 10^9^ CFU/mL.

### Effect of RamA on the MPC of ciprofloxacin

To elucidate the effect of RamA on the MPCs of the ST and SI2 strains to ciprofloxacin, the MPCs and mutant frequencies of ciprofloxacin against ST and SI2 in the presence, absence and overexpression of RamA were tested. Table 3 showed that the MPC of SR to ciprofloxacin was the same as that of ST. The MPCs of STRA to ciprofloxacin increased 5-fold compared to that of SR. Likewise, the MPCs of SI2R to ciprofloxacin decreased 3-fold compared to that of SI2 and its MSW became narrower. The overexpression of RamA in SI2R strain resulted in increased MPC 4-fold to ciprofloxacin.

**Table 3:** Mutant prevention concentration (MPC) of ciprofloxacin against the ST and SI2 strains in the presence, absence and overexpression of RamA.

Strains	Genotype	MIC (mg/l)	MPC (mg/l)	MPC/MIC
ST	wild-type	0.0125	0.3	24
SR	ST *ramA::aph*	0.0125	0.3	24
STRA	SR pGEXΦ(*gst-ramA*) +IPTG	0.05	1.5	30
SI2	Ser83→Phe in GyrA	0.4	6	15
SI2R	*ramA::aph*+Ser83→Phe in GyrA	0.3	2	7
SI2RA	SI2R pGEXΦ(*gst-ramA*) +IPTG	0.8	8	10

Notes: CIP, ciprofloxacin; ENR, enrofloxacin.

**Figure 3:**
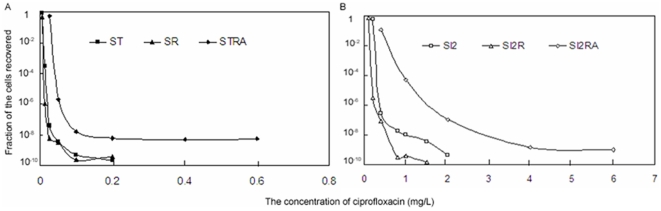
Mutation frequencies of the strains to ciprofloxacin. The SI2 strain was a spontaneous mutant harboring a Ser83→Phe mutation in the QRDR of GyrA from ST under the selection pressure of ciprofloxacin; the SR and SI2R strains were the mutant of ST*ramA::aph* and SI2*ramA::aph*, respectively; the STRA and SI2RA strains were the SR and SI2R with the overproduction of RamA, respectively.

Under the selection pressure of the same concentrations of ciprofloxacin, the mutant frequencies of the STRA and SI2RA strains with the overproduction of RamA all significantly augmented compared to that of the ST and SI2 strains, respectively (Figure 3). While the mutant frequencies of SI2R strain were lower than that of SI2 strain. Likewise, it was obvious that the overexpression of RamA promoted the development of ciprofloxacin resistant mutants from ST and SI2.

## Discussion

In this study, the two spontaneous mutants (SI1 and SI3) were selected from a susceptible strain of *S. enterica* serovars Typhimurium under the selection pressure of ciprofloxacin. The SI1 and SI3 strains were selected for further analysis since they demonstrated different resistance genotype to the tested drugs. The SI1 strain showed no target mutation in its QRDRs but exhibited decreased susceptibility to the tested drugs compared to the ST strain. On the other hand, although the SI3 strain demonstrated the same target site mutation as that of the SI2 strain, but it exhibited high resistance to fluoroquinolones as well as to tetracycline and chloramphenicol.

Our study demonstrated that RamA was the main factor that controlled the susceptibility of SI1 to ciprofloxacin by activating MdtK as well as increasing the expression level of *AcrAB*. Likewise, the accumulation experiment in SI1 indicated that at least an efflux pump independent on the electrochemical potential of H^+^ might exist and its efflux to ciprofloxacin depended on the RND efflux pump. It had been confirmed that AcrAB-TolC, a predominant efflux pump in the RND family depended on the electrochemical potential of H^+^ and it captured the substrates from the periplasm or the outer leaflet of the cytoplasmic membrane [41], [34]–[35]. While the MDR efflux pump MdtK belonged to the MATE family which mainly depended on the electrochemical potential of Na^+^ [42]. Recent report demonstrated that MdtK extruded fluoroquinolones into periplasmic space [43]. This study results indicated that the cooperation of AcrAB and MdtK might contribute to the efflux of ciprofloxacin in SI1. Interestingly, in *E.coli*, the high-level expression of *ydhE* (MATE family member) allowed the strain to grow on otherwise lethal concentrations of the fluoroquinolone norfloxacin and the cooperation of YdhE and AcrAB led to a synergic increase in fluoroquinolone resistance [44].

The second spontaneous mutant SI3 (CIP MICs, 16 mg/L) harboring a Ser83→Phe mutation in the QRDR of GyrA exhibited MDR. The result of ciprofloxacin accumulated in SI3 indicated the presence of at least another efflux pump that not belonging to the RND family but inhibited by CCCP. Genomic analyses revealed that *Salmonella* Typhimurium LT2 had five putative RND transporter systems, fourteen putative MF transporter systems, two putative MATE transporters systems and three putative ABC transporter systems (http://www.membranetransport.org). Among them, the members of RND, MF and MATE transporter systems could extrude quinolones when overexpressed in vitro [8]. It was reported that the member of MF family depended on the electrochemical potential of H^+^ and could extrude its substrate into periplasmic space or outer niche with the help of TolC [42], [43]. Therefore, the members in the MF family might contribute in the ciprofloxacin resistance in SI3.

AcrAB is a predominant MDR efflux pump in *S. enterica* serovars Typhimurium. Ricci et al. demonstrated that when AcrB was inactivated in a susceptible *S. enterica* serovars Typhimurium, the ciprofloxacin resistant mutants were very difficulty to be selected. When TolC was inactivated, no ciprofloxacin resistant mutant was selected [30]. Therefore, AcrAB was the main channel by which ciprofloxacin in the cell extrude directly into outer niche. In this study, the expression of RamA was mainly responsible for increasing the expression level of *acrAB*, which was corresponded to the previously published results [33], [45]. However, the expression level of *mdtK* in SI3 was higher than that in SI1 and STRA. The members of MF family might also contribute in the ciprofloxacin-resistance in SI3. These efflux pumps might extrude ciprofloxacin mainly into periplasmic space and then, AcrAB directly extrude the drug into outer niche. The synergetic transportation mechanism between AcrAB and other single component efflux pumps had been confirmed in *E.coli* [46], [47]. Likewise, our results also indicated that the synergetic transportation mechanism might exist in SI3. For instance, when RamA was overexpressed in SR or SI2R, the strains did not produce resistance to ciprofloxacin. When RamA was inactivated in SI3, the MICs of SI3R to ciprofloxacin dramatically decreased, whereas still higher than the resistant breakpoint described in CLSI [37]. On the other hand, the selected spontaneous mutants form SR (ST*ramA*::*aph*) under the selection pressure of different concentrations of ciprofloxacin exhibited decreased susceptibility to ciprofloxacin, whereas the same susceptibility to tetracycline and chloramphenicol as that in ST (unpublished data). As a result, the expression of RamA promoted the development of ciprofloxacin resistance by activating MdtK and increasing the expression level of *acrAB* in *S. enterica* serovars Typhimurium. Likewise, when MarA, another regulator of MDR efflux pumps was inactivated in SI1 and SI3, the susceptibility of the mutants to the drugs tested did not dramatically change (data not shown).

MPC and MSW focus on the development of resistant mutants in susceptible bacterial population. Since spontaneous mutants with a site mutation in gene usually arise at a frequency of about 10^−6^ to 10^−8^, the first-exposure to sub-lethal concentrations of fluoroquinolones screens mutants with a single target site mutation [48], [49]. In clinical practice, the presence of the first-step mutants in the bacterial population prior to fluoroquinolnes treatment may be the primary cause leading to therapeutic failure by the enrichment and amplification during therapy. In this study, the spontaneous mutants SI1 and SI2 were the first-step mutants from ST (the SI1 and SI2 strains were selected on 1×MIC and 8×MIC selecting plate, respectively). The results of MPC showed that the SI2RA strain with the overproduction of RamA had the highest MPCs among six strains tested and the MPC of SI2 was higher than that of STRA. Likewise, the mutant frequencies of SI2RA were higher than that of SI2 under the same selection pressure of ciprofloxacin. This indicated that the first-step mutants harboring a target site mutation in the QRDR of GyrA in a susceptible *S. enterica* serovars Typhimurium population were the main source for the development of ciprofloxacin resistant mutants. The overexpression of RamA promoted the development of ciprofloxacin resistant mutants. To be noticed, the MPC, MSW and mutant frequencies of the SI2R (SI2*ramA*::*aph*) strain to ciprofloxacin was all lower than that of SI2. Therefore, the inhibition of the expression level of *ramA* in *S. enterica* serovar Typhimurium could decrease the appearance of the second-step resistant mutants to ciprofloxacin.

RamR, a member of the TetR family is a local repressor of RamA. Inactivation of RamR in a susceptible *S. enterica* serovars Typhimurium strain exhibited decreased susceptibility to multidrugs due to the increased expression of AcrAB via the increased expression of RamA. In contrast, the inactivation of RamA, MarR, MarA, SoxR, and SoxS did not affect the susceptibilities of the strain [17]. Likewise, RamR could directly bind to the promoter region of *ramA* [50]. In this study, only mutations in *ramR* were found in the spontaneous mutants SI1 and SI3. When RamR was complemented in SI1, the MICs of SI1RR to fluoroquinolones decreased 2- to 4-fold compared to that of SI1, whereas were still higher that of ST. This indicated that these mutations in *ramR* participated in the ciprofloxacin resistance by increasing the expression level of *ramA*. Likewise, RamA was still regulated by other protein except for RamR in the development of ciprofloxacin resistance of *S. enterica* serovars Typhimurium. Currently, the changes of RamR were often found in clinical isolates of MDR *S. enterica* serovars Typhimurium and considered as the main cause leading to MDR of strain [18]. To the best of our knowledge, the mutations in RamR presented in this study have not been reported before (the nucleotide sequence of RamR found in the mutant SI1 has been assigned the accession number HQ114265 in the GenBank nucleotide sequence database).

In conclusion, RamA was the main factor that controlled the susceptibility of SI1 to ciprofloxacin by activating the MDR efflux pump MdtK as well as increasing the expression level of *acrAB*. In SI3, the expression of RamA was responsible for increasing the expression level of *acrAB*. In a susceptible *S. enterica* serovars Typhimurium population, the first-step mutants harboring a target site mutation in the QRDR of GyrA was the main source for the development of ciprofloxacin resistant mutants. The expression of RamA promoted the development of ciprofloxacin resistant mutants. The inhibition of RamA could decrease the appearance of the ciprofloxacin resistant mutants.
